# Centers for Disease Control and Prevention–Recognized Diabetes Prevention Program After Gestational Diabetes Mellitus

**DOI:** 10.1016/j.xagr.2022.100150

**Published:** 2022-12-17

**Authors:** Cassandra E. Henderson, Hasan Nezam, Katia Montserrat Castillo

**Affiliations:** 1 Teachers College, Columbia University, New York, NY (Dr Henderson); 2 Department of Obstetrics and Gynecology, Maternal and Fetal Medicine, Mercy Hospital, Rockville Centre, NY (Dr Henderson); 3 Environmental and Occupational Health Sciences Institute (EOHSI) - Rutgers University, Clinical Research and Occupational Medicine Piscataway, NJ (Dr Nezam); 4 Louisiana State University School of Medicine, Monroe Medical Center, Monroe, LA (Ms Montserrat Castillo)

**Keywords:** diabetes mellitus, diabetes mellitus in pregnancy, diabetes mellitus risk, Diabetes Prevention Program, disease prevention, gestational diabetes mellitus, obstetrics, pregnancy, recurrent gestational diabetes mellitus

## Abstract

Gestational diabetes mellitus is associated with an increased risk of developing type 2 diabetes mellitus. To decrease or delay the risk of developing type 2 diabetes mellitus after gestational diabetes mellitus, postpartum care should include a recommendation that the individual participates in a recognized Diabetes Prevention Program.

## Introduction

The background of prediabetes mellitus affecting 96 million people and the rising diabetes mellitus prevalence in the United States motivates us to advocate for the adoption of an evidence-based program to prevent diabetes mellitus in groups of individuals with a history of gestational diabetes mellitus (GDM). After a pregnancy complicated by GDM, during the immediate postpartum period or initial healthcare encounter, we suggest that the affected person participates in a Centers for Disease Control and Prevention (CDC)–recognized Diabetes Prevention Program (DPP) to decrease their risk of type 2 diabetes mellitus (T2D) and conceivably lower their risk of recurrent GDM.

## Background

In January of 2021, the CDC reported that of the US population, 37.3 million (11.3%) and 96 million (23%) people were living with diabetes or prediabetes mellitus, respectively.^1^ In 2001, Tuomilehto^2^ reported the results of the Finnish Diabetes Prevention Study that found that T2D could be prevented if high-risk patients adopted prescribed lifestyle changes. A 10-year follow-up of the adoption of these lifestyle changes was evaluated in the National Institute of Diabetes and Digestive and Kidney Diseases–sponsored CDC DPP that was a randomized, controlled clinical trial conducted at 27 US clinical centers enrolling >3230 individuals. This trial demonstrated that individuals at high risk for T2D, who achieve significant weight reduction by participating in a lifestyle modification program (through increased physical activity and dietary changes), could prevent or postpone the onset of T2D.^3^

## Lifestyle modification

The CDC DPP trial demonstrated that lifestyle modification reduced the chances of developing T2D by 60% when compared with those in the placebo group. The study groups included individuals at high risk for developing T2D by virtue of having had GDM.^4^ Several other studies using the DPP model demonstrated that a 7% weight reduction via lifestyle modification could significantly decrease the risk of developing T2D. DPP has been found to be an effective technique to induce behavioral changes and weight reduction, and reduce cardiometabolic risk factors in general, especially for individuals with a history of GDM.^5^

## Gestational diabetes mellitus and type 2 diabetes mellitus risk

GDM is any form of glucose/carbohydrate intolerance with first onset or recognition during pregnancy. The US Preventive Services Task Force recommends routine antenatal glucose screening for GDM between 24 and 26 weeks of gestation. Two to 10% of pregnancies in the United States are affected by GDM, which confers a 35% to 60% risk of developing T2D during the subsequent 10 to 20 years.^6^

## Referral to the National Diabetes Prevention Program

The link https://dprp.cdc.gov/Registry can be used to access the national registry of >2000 CDC-recognized DPPs and their contact information. Several reports have documented numerous barriers to attending postnatal clinical appointments and program interventions. The most cited barriers to participation include inaccurate patient contact information, lack of child care, work or school obligations, and lack of access to transportation.^7^^,^^8^ Participation in postpartum DPPs would likely be hampered by similar barriers. To overcome these potential barriers, we suggest that the increased number of remote, distance, or online CDC-recognized DPPs created in response to the pandemic-related change in healthcare delivery might mitigate or remove some of the obstacles and thus facilitate participation in postpartum DPPs.

## Discussion

Many medical organizations including the American Diabetes Association have encouraged healthcare practitioners (HCPs) to refer their high-risk patients to a lifestyle-change program, such as the one offered through the National DPP. A recent study showed that HCPs who were familiar with lifestyle-change DPPs and aware of available programs were more likely to make DPP referrals. There is also evidence that patients who were referred to a lifestyle-change program by their HCP were more likely to join the program. Unfortunately, according to the CDC, 80% of patients with prediabetes mellitus have no knowledge of their diagnosis, and only 5% of patients with prediabetes mellitus or at high risk of T2D receive referrals to a program for lifestyle change.^9^ Currently, after a GDM-affected pregnancy, to evaluate persistent or recurrent glucose intolerance, the postdelivery recommendation is to perform an oral glucose tolerance test (OGTT) between 4 and 12 weeks after delivery and subsequent serial OGTTs at 1-to-3-year intervals.^10^

Up to 70% of individuals with GDM will develop T2D in the absence of intervention.^6^ GDM is associated with higher rates of preeclampsia, cesarean delivery, fetal macrosomia, neonatal hypoglycemia, hyperlipidemia, shoulder dystocia, birth trauma, and stillbirth. Moreover, the offspring from GDM-affected pregnancies have an increased risk for childhood and adult-onset obesity.^11^^,^^12^ Decreasing the risk of T2D and perhaps the recurrence of GDM is a desirable and realizable DPP goal for public health.

## Conclusion

To decrease the GDM-associated risk of developing T2D, routine postpartum care should include a recommendation that the affected individual participates in a CDC-recognized DPP.
